# MAPK CcSakA of the HOG Pathway Is Involved in Stipe Elongation during Fruiting Body Development in *Coprinopsis cinerea*

**DOI:** 10.3390/jof8050534

**Published:** 2022-05-20

**Authors:** Jing Zhao, Jing Yuan, Yating Chen, Yu Wang, Jing Chen, Jingjing Bi, Linna Lyu, Cigang Yu, Sheng Yuan, Zhonghua Liu

**Affiliations:** 1 Jiangsu Key Laboratory for Microbes and Microbial Functional Genomics, Jiangsu Engineering and Technology Research Center for Industrialization of Microbial Resources, College of Life Science, Nanjing Normal University, Nanjing 210023, China; 181202108@njnu.edu.cn (J.Z.); 201202039@njnu.edu.cn (J.Y.); 201202040@njnu.edu.cn (Y.C.); 191202036@njnu.edu.cn (Y.W.); 211202037@njnu.edu.cn (J.C.); 191202110@njnu.edu.cn (J.B.); llnlln1207@163.com (L.L.); yuansheng@njnu.edu.cn (S.Y.); 2 Nanjing Institute of Environmental Sciences, Ministry of Ecology and Environment, Nanjing 210042, China

**Keywords:** HOG pathway, mitogen-activated protein kinase, fruiting body development, stipe elongation

## Abstract

Mitogen-activated protein kinase (MAPK) pathways, such as the high-osmolarity glycerol mitogen-activated protein kinase (HOG) pathway, are evolutionarily conserved signaling modules responsible for transmitting environmental stress signals in eukaryotic organisms. Here, we identified the MAPK homologue in the HOG pathway of *Coprinopsis cinerea,* which was named CcSakA. Furthermore, during the development of the fruiting body, CcSakA was phosphorylated in the fast elongating apical part of the stipe, which meant that CcSakA was activated in the apical elongating stipe region of the fruiting body. The knockdown of CcSakA resulted in a shorter stipe of the fruiting body compared to the control strain, and the expression of phosphomimicking mutant CcSakA led to a longer stipe of the fruiting body compared to the control strain. The chitinase CcChiE1, which plays a key role during stipe elongation, was downregulated in the CcSakA knockdown strains and upregulated in the CcSakA phosphomimicking mutant strains. The results indicated that CcSakA participated in the elongation of stipes in the fruiting body development of *C. cinerea* by regulating the expression of CcChiE1. Analysis of the H_2_O_2_ concentration in different parts of the stipe showed that the oxidative stress in the elongating part of the stipe was higher than those in the non-elongating part. The results indicated that CcSakA of the HOG pathway may be activated by oxidative stress. Our results demonstrated that the HOG pathway transmits stress signals and regulates the expression of CcChiE1 during fruiting body development in *C. cinerea*.

## 1. Introduction

The development of the fruiting body of basidiomycetes is a highly complex process controlled by environmental, genetic, and physiological factors [1,2]. Environmental conditions play a crucial role in the formation and morphogenesis of fruiting bodies [3,4,5]. However, it is still unclear how the fungus senses environmental factors during the development of the fruiting body. *Coprinopsis cinerea* is an edible fungus that is used as a model fungus to study the fruiting body development mechanism of basidiomycetes [6,7]. The fruiting body development of *C. cinerea* can be divided into five stages: (1) the formation of the primary hyphal knot; (2) the formation of the secondary hyphal knot; (3) the formation of the primordia; (4) the elongation of the stipe; and (5) the opening and autolysis of the pilei and the maturation and release of basidiospores [2,8]. To initiate and continue fruiting body development, several environmental signals are required, such as light-dark periods, low nutrition, appropriate temperature, and suitable CO_2_ concentrations [3,4,7,9]. Under the control of environmental factors, a range of genes are expressed to participate in the morphogenesis of the fruiting body. For example, *Arp9*, *Pcc1*, and *Ubc2* are involved in clamp cell formation and are essential for fruiting initiation [10,11,12]. *Cag1* is preferentially expressed in gill trama tissue cells and is involved in the formation of the pileus during the development of primordia [13]. *Eln2* participates in the development of primordia and stipe elongation [14]. Transcription of *Eln3* is specifically activated in the rapidly elongating stipe and participates in stipe elongation [15]. The *Ich1* gene is specifically expressed in the pileus of the fruiting body and is essential for pileus formation [16]. The transcription of *Exp1* is strongly induced in the pileus before pileus expansion and essential for pileus expansion and autolysis [17]. Our previous studies show that some glycoside hydrolases participate in the morphogenesis of the fruiting body [6]. The chitinases *ChiE1* and *ChiIII*, chitin deacetylases *Cda1* and *Cda2*, glucosidase *Bgl2* and glucanase *Eng16A* are highly expressed in the stipe and are involved in the elongation of the stipe [18,19,20]. The chitinases *ChiB1*, *ChiEn1*, and *ChiIII*, glucosidase *Bgl1*, and glucanases *Eng* and *Exg* are highly expressed in the pileus and participate in the autolysis of the pileus [8,18,21,22,23]. However, it is still not entirely clear how the above genes are regulated by *C. cinerea* in response to environmental factors during fruiting body development. Some studies show that photoreceptors for blue light, such as Dst1, Dst2, and WC-2, may sense light signals in the environment and are involved in fruiting body photomorphogenesis of *C. cinerea*, but their downstream regulatory genes are not yet known [4,9,24]. MAPK pathways are evolutionarily conserved signaling modules in eukaryotic organisms [25]. In fungi such as *Saccharomyces cerevisiae* and *Aspergillus fumigatus*, the MAPK pathways respond to a variety of environmental signals, such as cell wall stress, light, nitrogen, and carbon deprivation and high osmolarity, and regulate the expression of genes associated with cell differentiation and development [25,26,27,28,29,30].

However, the physiological function of the MAPK pathways in the fruiting body development of basidiomycetes has not been elucidated. In this study, the phosphorylation levels of MAPK during fruiting body development of *C. cinerea* were analyzed. Among them, only the kinase of the high-osmolarity glycerol mitogen-activated protein kinase pathway (HOG pathway) showed different levels of phosphorylation in the stipe of the fruiting body. Therefore, the kinase of the HOG pathway was further analyzed to expose its physiological functions during fruiting body development.

## 2. Materials and Methods

### 2.1. Strains and Cultures

*C. cinerea* strain AmutBmut (*A43mut B43mut pab1-1*) was purchased from the Japan Collection of Microorganisms (JCM, Ibaraki, Japan). For cultivation of the AmutBmut strain or transformants of AmutBmut, an agar block with mycelium was inoculated in the center of PDYA medium agar in Petri dishes, 7 cm in diameter and incubated at 28 °C in constant darkness in an incubator for 4 days until the mycelia covered the entire medium surface; then, the mycelia on the Petri dishes were transferred at 28 °C to a 12 h light/12 h dark rhythm condition (50 μmoles/m^2^/s white light from LED lamps, Ruihua, Wuhan, China) or to constant darkness in the incubator to continuously grow for the indicated number of days [7,8].

### 2.2. Construction of Plasmids and DNA Transformation

The plasmids pCc*pab-1* and pCcExp were constructed by our laboratory in a previous study [19]. For construction of the gene silencing plasmid, the antisense fragment (601 to 101 bp) and sense fragment (101 to 601 bp) of CcSakA were amplified by PCR from the cDNA of *C. cinerea* and ligated into the *NcoI* and *KpnI* sites of pCcExp, respectively, to generate plasmid pCC-SakAi. For construction of the phosphomimicking CcSakA mutant expression plasmid, the gDNA fragment of CcSakA with mutation sites was amplified by overlap PCR and ligated into the pCcExp between the *NcoI* and *KpnI* sites to generate plasmid pCC-SakA^T170E+Y172D^. DNA transformation experiments were performed as previously described [19,20]. pCC-SakAi and pCc*pab-1* were cotransformed into the *C. cinerea* strain AmutBmut to generate the knockdown transformant SakAi. The plasmids pCC-SakA^T170E+Y172D^ and pCc*pab-1* were cotransformed to generate the CcSakA phosphomimicking mutant transformant SakAm. pCcExp and pCc*pab-1* were cotransformed to generate mock transformants.

### 2.3. Protein Extraction and Western Blotting

The apical, median, and basal 1 cm regions of the 6 cm stipe of *C. cinerea* were harvested and ground thoroughly in liquid nitrogen. The ground powder was homogenized in prechilled lysis buffer (50 mM Tris-HCl pH 7.5, 150 mM NaCl, 5 mM EDTA, and 1× protease and phosphatase inhibitor cocktail for fungal and yeast extracts (Beyotime, China)) with a Bioprep 24 homogenizer (Allsheng, Hangzhou, China) (5 × 30 s at 3000 rpm) in the presence of glass beads (ø = 0.5 mm). The suspensions were centrifuged at 4 °C and 12,000× *g* for 10 min. Subsequently, 50 μg protein samples were separated on a 12% SDS–PAGE gel and transferred to polyvinylidene difluoride (PVDF) membranes (Millipore, Darmstadt, Germany) by using a Trans-Blot semidry transfer unit (Bio–Rad, Hercules, CA, USA), as described previously [19]. Blots for phosphorylated CcSakA were probed with rabbit antiphospho-p38 MAPK antibody (Thr180/Tyr182, cat. 9211; Cell Signalling, Danvers, MA, USA) [31,32]. Bound primary antibodies were revealed using horseradish peroxidase (HRP)-conjugated goat anti-rabbit antibody (Sangon Biotech, Shanghai, China). Western blots were developed using the Enhanced ECL Chemiluminescence Detection Kit (Vazyme, Nanjing, China), and images were collected with the Tanon-5200 chemiluminescent imaging system (China) [19]. β-tubulin was used as a loading control with anti-β-tubulin monoclonal antibody (Solarbio, Beijing, China) as the primary antibody and HRP-conjugated goat anti-mouse antibody (Sangon Biotech, Shanghai, China) as the secondary antibody.

### 2.4. qRT–PCR Analysis

Total RNA was extracted from the apical region of the stipe using the Spin Column Fungal Total RNA Purification Kit (Sangon Biotech, Shanghai, China). First-strand cDNA was synthesized from total RNA using the HiScript II Q RT Supermix for qPCR Kit (+gDNA wiper) (Vazyme, Nanjing, China), and quantitative real-time PCR (qRT–PCR) analysis was conducted using a pair of specific primers for each gene (Table S1) and AceQ qPCR SYBR Green Master Mix (Vazyme, Nanjing, China). The gene expression levels were normalized to β-tubulin, and the fold expression of target genes relative to β-tubulin was calculated according to the 2^−ΔΔCT^ method [8].

### 2.5. Osmolality Analysis

The osmolality of the different regions of the stipe was analyzed according to the method reported by Paljakka et al. [33,34], with appropriate modifications. The apical, median, and basal 1 cm regions of the 6 cm stipe of *C. cinerea* were harvested, weighed as fresh weight (FW), and frozen in a sealed cryotube under liquid nitrogen. After being frozen for 24 h, the samples were removed and dried to a constant weight in an oven at 70 °C for 72 h to obtain the dry weight (DW). To measure the turgid weight (TW), fresh samples were saturated in closed tubes with Milli-Q water at 4 °C for 48 h, and then the samples were weighed as TW after the water on the surface of the samples was wiped carefully. The relative water content (RWC) was calculated as

(1) RWC=(FW−DW)/(TW−DW)

To measure the in-situ osmolality (osMol_in situ_), the frozen samples were thawed inside the closed tubes at 25 °C for 1 h. T samples were then set in silica-based membrane collection tubes (Sangon Biotech, Shanghai, China) and centrifuged at 4 °C and 12,000× *g* for 15 min. The extracted liquid was immediately measured with a freezing point osmometer (Fiske Model 110, Washington, USA) as osMol_in situ_. The osmolality at full saturation osMol_full saturation_ was calculated as

(2) $${\mathrm{osMol}}_{\mathrm{full}\;\mathrm{saturation}}={\mathrm{osMol}}_{\mathrm{in}\;\mathrm{situ}}\times \mathrm{RWC}$$

### 2.6. H_2_O_2_ and ROS Measures

To analyze the H_2_O_2_ concentrations of the different regions of the stipe, the tissue fluid was extracted according to the method described previously. The H_2_O_2_ concentration of the extracted liquid was analyzed using a hydrogen peroxide assay kit (Jiancheng, Nanjing, China) [35]. The protein concentration was analyzed using a total protein assay kit (Jiancheng, Nanjing, China), according to the method described in the instruction. For the fluorescence assay, the different regions of the stipe were stained with 2′, 7′-dichlorodihydrofluorescein diacetate (DCFH-DA, Beyotime, Shanghai, China) to investigate intracellular reactive oxygen species (ROS) [36,37]. Briefly, the different regions of the stipe were sliced and incubated with DCFH-DA for 20 min at 37 °C, and then washed with Tris-HCl buffer (50 mM, pH 7.5). The stained tissues were observed using fluorescence microscopy (Olympus) and measured at 488 nm excitation and 525 nm emission. Nine random sights were selected to analyze the fluorescence intensity by using ImageJ 1.51 [36].

### 2.7. Chitinase Activity Analysis

To analyze the chitinase activity of stipe, the protein was extracted according to the growing apical region of stipe to the method described previously. The chitinase activity of the supernate was determined as described by Zhou et al. [19]. One unit of chitinase activity was defined as the amount of enzyme that liberates the reducing sugar, corresponding to 1 μmol of N-acetylglucosamine per min [19].

To analyze the chitinase activity of stipe, the protein was extracted according to the growing apical region of stipe to the method described previously. The chitinase activity of the supernate was determined as described by Zhou et al. [19]. One unit of chitinase activity was defined as the amount of enzyme that liberates the reducing sugar, corresponding to 1 μmol of N-acetylglucosamine per min [19].

### 2.8. Statistical Analysis

Tests for significant differences were carried out by performing paired t-tests in Microsoft Excel 2010 or Duncan’s multiple range test (significance set at 0.05) in SPSS Statistics 17.0.

## 3. Results

### 3.1. Identification of the MAPK of the HOG Pathway in C. cinerea and Its Phosphorylation in Stipes during Fruiting Body Development

**Figure 1:**
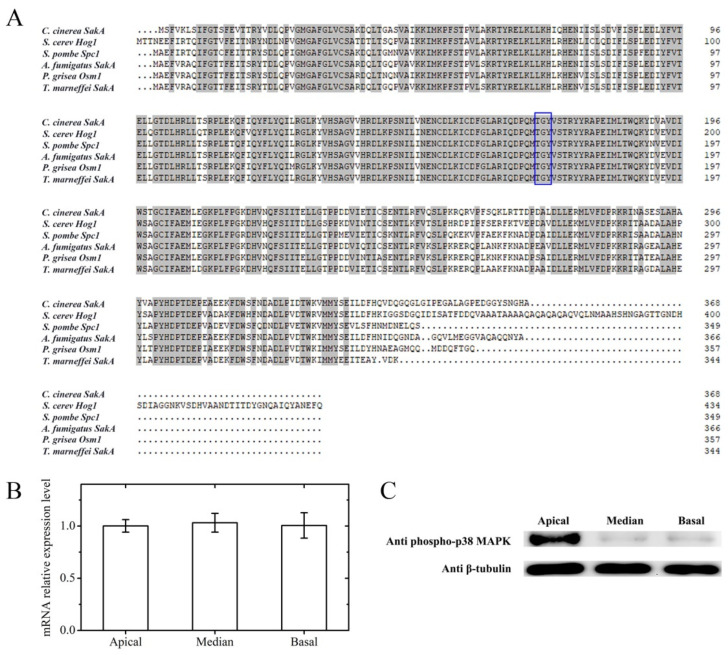
(**A**) The *CcsakA* gene encodes a putative homologue of the MAPK of the HOG pathway in *C. cinerea*. The amino acid sequence of CcSakA is aligned with *S. cerevisiae* Hog1 [40], *S. pombe* Spc1 [41], *A. fumigatus* SakA [39], *P. grisea* Osm1 [42], and *T. marneffei* SakA [43]. Conserved TGY phosphorylation sites found in the stress Hog1/Spc1/p38 MAPK family are marked with blue boxes. (**B**) The relative mRNA expression level of *sakA* in the different stipe regions during the development of *C. cinerea* fruiting bodies. (**C**) Western blotting of the phosphorylation of CcSakA in the extracts from different stipe regions during the development of *C. cinerea* fruiting bodies. Phosphorylated CcSakA was detected using anti-phospho-p38 MAPK antibody. Levels of β-tubulin were used to demonstrate equal protein loading.

Based on the protein sequences of Hog1 (NC_001144.5) in *S. cerevisiae* and SakA (XP_752664.1) in *A. fumigatus* in the National Center for Biotechnology Information (NCBI) database, a putative homologue of the MAPK of the HOG pathway in *C. cinerea* (XP_001829398.2) was identified by BLASTP. The corresponding gene was named *CcsakA* (*C. cinerea* stress activated kinase A; Gene ID: 6005827; Gene symbol: CC1G_00577). CcSakA was described as a CMGC/MAPK protein kinase in GenBank. Homology analysis showed that the CcSakA protein sequence consisted of 368 amino acid residues and had 80.22% identity to SakA from *A. fumigatus*. Compared to Hog1 in *S. cerevisiae*, Spc1 in *Schizosaccharomyces pombe*, Osm1 in *Pyricularia grisea*, and SakA in *Talaromyces marneffei*, similarity among these MAPKs was extended along the entire polypeptide, including the conserved TGY phosphorylation site found in the stress Hog1/Spc1/p38 MAPK family (Figure 1A) [38,39]. In the fruiting body of *C. cinerea*, the relative mRNA expression level of *sakA* was not significantly different in different regions of stipe (Figure 1B). To detect whether the HOG pathway is activated during the fruiting body development of *C. cinerea*, protein extracts were obtained from the fast elongating apical part, the slow elongating median part, and the non-elongating basal part of the stipe and analyzed by Western blotting with an anti-phospho-p38 MAPK antibody. When the anti-phospho-p38 MAPK antibody was used to probe the phosphorylation of CcSakA, a strong band of approximately 43 kDa was detected in the apical part of the stipe, but the bands of the same size in the extracts of the median and basal parts were very weak (Figure 1C). The results showed that the expression levels of *sakA* in the different regions of stipe were not different. However, the phosphorylation levels of the protein were significantly higher at the apical part of stipe than that at the median and basal parts.

### 3.2. Effects of dsRNA-Induced Silencing of CcSakA on the Stipe Elongation of C. cinerea

**Figure 2:**
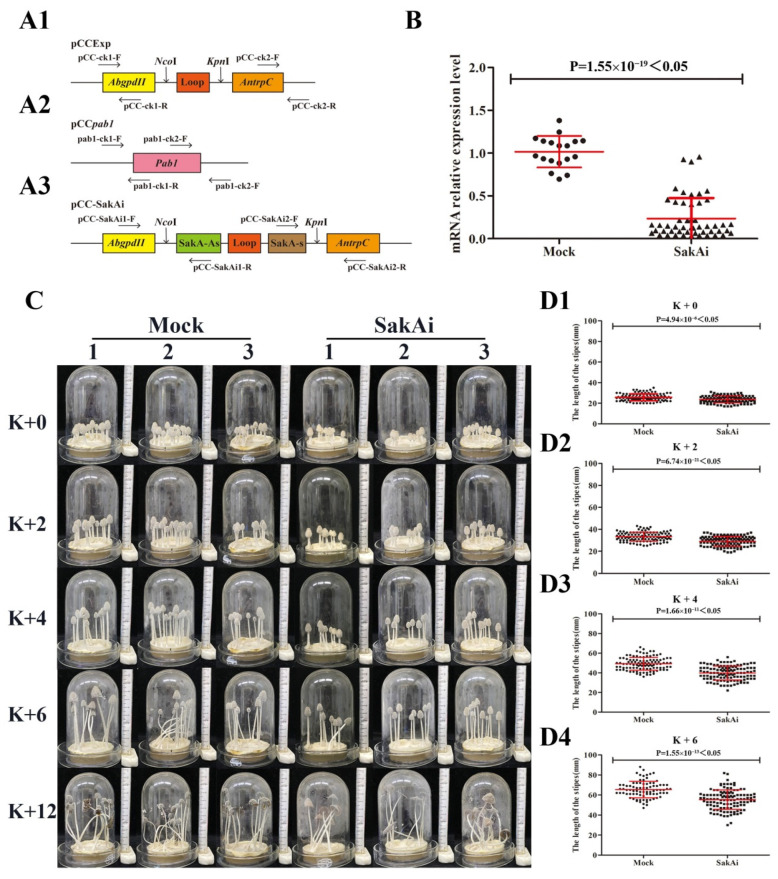
Construction and phenotypes of CcSakA gene-silenced strains. (**A**) Schematic representation of plasmids pCcExp (**A1**), pCc*pab-1* (**A2**), and pCC-SakAi (**A3**). The arrows below the plasmids indicate the primers for genomic PCR. (**B**) The relative mRNA expression level of *sakA* in mock transformants (circle) and SakAi transformants (triangle). (**C**) Growing fruiting bodies of the three representatives of the mock transformants and SakAi transformants at different time points. (**D**) The stipe lengths of the fruiting bodies of four mock transformants (circle, *n* = 109 fruiting bodies) and eight SakAi transformants (square, *n* = 124 fruiting bodies) with at least three repeats of each transformant at K + 0 (**D1**), K + 2 (**D2**), K + 4 (**D3**), and K + 6 (**D4**).

Because targeted gene disruption is particularly intractable in *C. cinerea*, a double-stranded RNA (dsRNA)-mediated gene silencing strategy was used to silence CcSakA in this study [19,44,45]. The plasmid pCC-SakAi (Figure 2(A3)) was constructed and cotransformed into the haploid oidia of the *C. cinerea* homothallic strain AmutBmut with the marker plasmid pCc*pab-1* (Figure 2(A2)) to generate the knockdown transformants SakAi. The empty plasmids pCcExp (Figure 2(A1)) and pCc*pab-1* were cotransformed into haploid oidia to generate mock transformants. More than 10 SakAi transformants were confirmed by genomic PCR, and eight of these were randomly selected for phenotype analysis. qRT–PCR analysis showed that the expression of CcSakA was 76.8% lower in the SakAi transformants than in the mock transformants (Figure 2B). When the transformants were inoculated in the center of the PDYA medium agar in Petri dishes 7 cm in diameter by using a 5 mm diameter hole punch and incubated at 28 °C in darkness for 96 h, the mycelial transformants of the mock strains and SakAi strains covered the entire agar medium surface. The transformants were then incubated under a 12 h light/12 h dark rhythm at 28 °C for an extra 6–7 days to produce fruiting bodies (Figure 2C). The results showed that the height of fruiting bodies of SakAi transformants was lower than that of the mock transformants. The time point at which the last light incubation ended and the dark incubation began was marked as K + 0. The time points 2 h, 4 h, 6 h, and 12 h after K + 0 were marked as K + 2, K + 4, K + 6, and K + 12 (Figure 2C). At K + 0, K + 2, K + 4, and K + 6, the average heights of fruiting bodies of the mock transformants were 25.72, 33.18, 49.20, and 65.48 mm, respectively (Figure 2D). However, the average heights of fruiting bodies of the SakAi strains at the corresponding time points were 23.71, 29.08, 39.86, and 55.35 mm, which were 7.81%, 12.36%, 18.98%, and 15.47% less than those in the mock transformants (Figure 2D).

### 3.3. Effects of Expression of a Phosphomimicking Mutant CcSakA on the Stipe Elongation of C. cinerea

**Figure 3:**
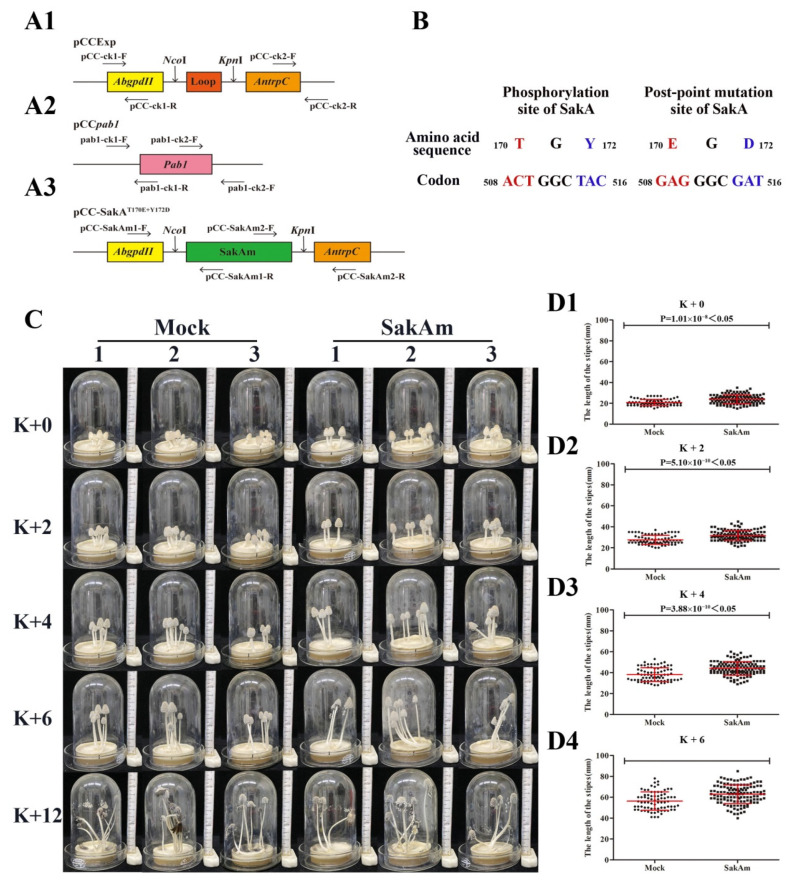
Construction and phenotypes of CcSakA phosphomimicking mutant strains. (**A**) Schematic representation of plasmids pCcExp (**A1**), pCc*pab-1* (**A2**), and pCC-SakA^T170E+Y172D^ (**A3**). The arrows below the plasmids indicate the primers for genomic PCR. (**B**) Schematic diagram of SakA phosphorylation site mutations. (**C**) Growing fruiting bodies of the three representatives of the mock transformants and SakAm transformants at different time points. (**D**) The stipe lengths of the fruiting bodies of four mock transformants (circle, *n* = 123 fruiting bodies) and eight SakAm transformants (square, *n* = 131 fruiting bodies) with at least three repeats of each transformant at K + 0 (**D1**), K + 2 (**D2**), K + 4 (**D3**), and K + 6 (**D4**).

The activation of CcSakA, which is the MAPK in the HOG pathway, was dependent on its phosphorylation on the threonine residue and tyrosine residue in the conserved TGY phosphorylation site [39,46]. To further analyze the role of CcSakA in the mycelium growth and stipe elongation of *C. cinerea*, the phosphomimicking mutant of CcSakA bearing a T170E/Y172D substitution within the TGY dual phosphorylation motif was constructed [47]. The plasmid pCC-SakA^T170E+Y172D^ (Figure 3(A3)) for the expression of the phosphomimicking mutant of the CcSakA mutant was constructed and cotransformed into the haploid oidia of the *C. cinerea* homothallic strain AmutBmut with the marker plasmid pCc*pab-1* (Figure 3(A2)) to generate the CcSakA phosphomimicking mutant transformant SakAm. More than 10 SakAm transformants were confirmed by genomic PCR, and eight of these were randomly selected for phenotype analysis. When the SakAm transformants and mock transformants were inoculated in the center of the PDYA medium agar in Petri dishes 7 cm in diameter by using a 5 mm diameter hole punch and incubated at 28 °C in darkness for 96 h, the transformants were incubated under a 12 h light/12 h dark rhythm at 28 °C for an extra 6–7 days to produce fruiting bodies (Figure 3C). At K + 0, K + 2, K + 4, and K + 6, the average heights of fruiting bodies of the mock transformants were 21.43, 27.81, 38.56, and 56.90 mm, respectively (Figure 3D). Furthermore, the average heights of fruiting bodies of the SakAm strains were 23.98, 31.73, 44.08, and 62.92 mm at K + 0, K + 2, K + 4, and K + 6, which were 11.90%, 14.20%, 14.32%, and 10.58% higher than those in the mock transformants at the corresponding time points (Figure 3D).

### 3.4. Gene Silencing or Point Mutation of CcSakA Affected the Expression of Chitinase CcChiE1 in C. cinerea

**Figure 4:**
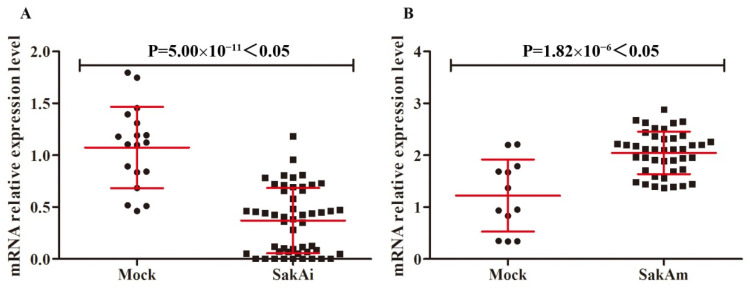
qRT–PCR analysis of the expression level of chitinase CcChiE1 in CcSakA gene silencing (SakAi) transformants (**A**) and CcSakA phosphomimicking mutant (SakAm) transformants (**B**) compared with mock transformants. A β-tubulin gene was used to standardize the mRNA level. Four mock transformants (circle), eight SakAi transformants (square in (**A**)), and eight SakAm transformants (square in (**B**)) were analyzed with at least three repeats of each transformant. Data are presented as the mean and standard error. Tests for significance were performed by a t-test in Microsoft Excel 2010.

In this study, the expression of a series of enzymes with cell wall synthesis and remodelling in *C. cinerea* were analyzed by qRT–PCR of CcSakA gene silencing (SakAi) transformants and CcSakA phosphomimicking mutant (SakAm) transformants, including chitin synthetases, glucan synthases, chitinases, and glucanases. The experimental results showed that only the expression of chitinase CcChiE1 differed significantly in different transformants. In the SakAi transformants, the expression level of CcChiE1 was 65.54% lower than that in the mock transformants (Figure 4A). In contrast, the expression level of CcChiE1 in the SakAm transformants was 71.42% higher than that in the mock transformants (Figure 4B). The chitinase activity in the apical region stipe of CcSakA gene silencing (SakAi) transformants was 4.00 × 10^−2^ U/mg, which was 16.31% lower than that of mock transformants. The chitinase activity of CcSakA phosphomimicking mutant (SakAm) transformants was 5.13 × 10^−2^ U/mg, which was 7.44% higher than that of mock transformants (Figure S1).

### 3.5. Oxidative Stress Was Higher in the Apical Part of the Stipe Than in the Median and Basal Parts

**Figure 5:**
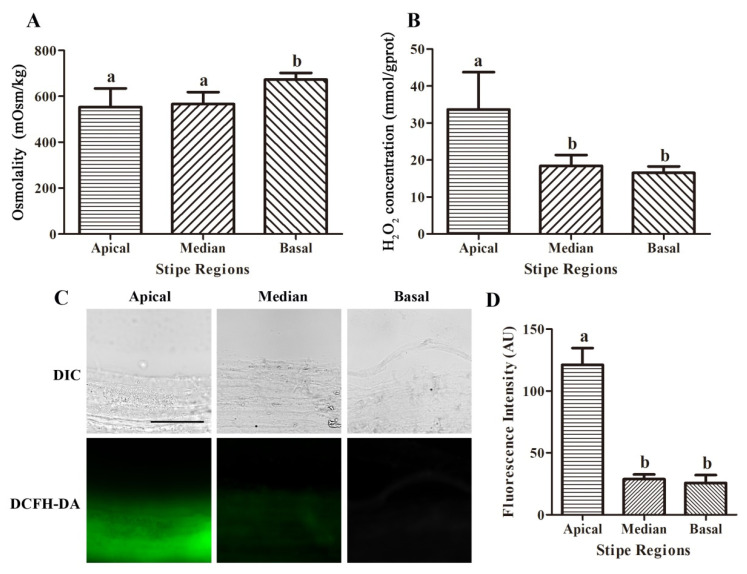
The osmolality (**A**) and H_2_O_2_ concentration (**B**) in the fast elongating apical part, the slow elongating median part, and the nonelongating basal part of the stipe of *C. cinerea*. Data are presented as the mean and standard error of three biological replicates (*n* = 6). The same letters indicate no significant difference (*p* > 0.05), and different letters indicate significant differences (*p* < 0.05) by Duncan’s test. The intracellular ROS imaging of the different regions of the stipe (**C**). The fluorescence signals of the fluorescent intracellular ROS probe DCFH-DA were observed and Bar = 50 μm. The fluorescence intensity (**D**) was analyzed by using ImageJ 1.51 (*n* = 9). The same letters indicate no significant difference (*p* > 0.05), and different letters indicate significant differences (*p* < 0.05) by Duncan’s test.

The HOG pathway of fungi is activated in the event of high osmotic stress or oxidative stress, and MAPKs (such as Hog1 in *S. cerevisiae* and SakA in *A. nidulans*) in the HOG pathway are phosphorylated [39,48]. Our results showed that the phosphorylation level of CcSakA of *C. cinerea* in the apical part of the stipe was significantly higher than that in the middle and basal parts of the stipe. Therefore, the levels of osmotic stress and oxidative stress in different parts of the stipe were analyzed by detecting the osmolality and H_2_O_2_ concentration in the fast elongating apical part, the slow elongating median part, and the nonelongating basal part of the stipe of *C. cinerea*. The results showed that the osmolality in the apical part of the stipe was 553.3 mOsm/kg, and the osmolality in the median part was 556.3 mOsm/kg, which was not significantly different from the apical part (Figure 5A). However, the osmolality in the basal part of the stipe was 672.9 mOsm/kg, which was 21.62% higher than that in the apical part and 18.78% higher than that in the median part (Figure 5A). The concentration of H_2_O_2_ in the apical part was 33.70 mmol/gprot, the concentration of H_2_O_2_ in the median part was 18.44 mmol/gprot, and the concentration of H_2_O_2_ in the basal part was 16.52 mmol/gprot (Figure 5B). The concentration of H_2_O_2_ in the apical part was 82.75% higher than that in the median part and 104.0% higher than that in the basal part (Figure 5B). Furthermore, DCFH-DA, an intracellular ROS fluorescent probe, was used, and the fluorescence was analyzed (Figure 5C). The results showed that the fluorescence intensity of the apical part was 302.3% higher than that of the median part and 371.6% higher than that of the basal part (Figure 5D), indicating a higher level of ROS in the apical part of stipe than in the median part and basal part.

## 4. Discussion

The fruiting body of Basidiomycetes is triggered by the induction of environmental stresses, including low nutrient, low temperature, and light conditions [49]. During the development of the fruiting body, fungal cells begin to differentiate and form mature fruiting bodies in response to physical signals (light, temperature, gravity, humidity) and chemical signals from the environment [50]. However, how Basidiomycetes sense different environmental stresses during fruiting body development is still unclear [49]. In yeasts and filamentous fungi, MAPK cascades are important signaling pathways to respond to environmental stresses and regulate processes, such as the cell cycle, reproduction, cell differentiation, morphogenesis, and stress response [25,28,30,51,52,53,54]. The signal induced by environmental stresses is transmitted by the sequential phosphorylation of a basic array of three proteins, often termed MAPKKK, MAPKK, and MAPK, [54,55]. The HOG pathway is highly conserved in fungi. In the basidiomycete *Sporisorium scitamineum,* MAPK SsHog1 is involved in the oxidative stress response [56]. In *Piriformospora indica*, PiHOG1 is involved in the salinity response [57]. In *Ganoderma lucidum*, the phosphorylation of Hog1 was enhanced when the mycelium was treated with oxidative stress [58]. However, the physiological function of the HOG pathway in the fruiting body development of basidiomycetes has not been elucidated. In this study, we identified the MAPK homologue in the HOG pathway of *C. cinerea*, which was named CcSakA. Furthermore, CcSakA was phosphorylated in the apical part of the stipe of the fruiting body, which meant CcSakA was activated in the apical elongating stipe region of the fruiting body. The knockdown of CcSakA resulted in a shorter stipe of the fruiting body compared to the control strain, and the expression of phosphomimicking mutant CcSakA led to a longer stipe of the fruiting body compared to the control strain. We presume that CcSakA mainly functions in the elongation of stipes during fruiting body development.

*C. cinerea* is one of the model basidiomycetes that has multiple developmental pathways [2,3,59]. During stipe elongation of the fruiting body, a series of glycoside hydrolases are involved in the cell wall remodeling of stipes [6,18,19,60,61]. Among them, chitinase ChiE1 (XP_001841026.2), which has stipe wall extension activity, plays a key role in stipe elongation growth during the development of the fruiting body and is highly expressed in the growing apical stipe region [6,19]. In this study, the expression level of the above glycoside hydrolases was examined in CcSakA knockdown strains, CcSakA phosphomimicking mutant strains, and mock strains (data not shown except CcChiE1). Of these, only the expression of CcChiE1 showed significant differences between strains. CcChiE1 was downregulated in the CcSakA knockdown strains and upregulated in the CcSakA phosphomimicking mutant strains. The results indicated that CcSakA participated in the elongation of stipes during fruiting body development by regulating the expression of CcChiE1. However, we presume that the expression of CcChiE1 may be indirectly regulated by CcSakA. In future studies, the downstream regulatory pathway of CcSakA will be investigated.

In fungi, the HOG pathway is involved not only in the response to osmotic pressure but also in the response to UV, light, heavy metal, heat, citric acid, and oxidative stresses [25,28,58,62,63,64]. Since the stipe would not continue to elongate normally after being dissected from the fruiting body of *C. cinerea* and because of the presence of a hydrophobic material layer outside the stipe, it was not possible to treat the stipe with solutions containing different stresses [7,59]. Therefore, to analyze which stresses the HOG pathway responds to during the development of the fruiting body, the osmolality and H_2_O_2_ concentration in different parts of the stipe were analyzed. However, the osmolarity in the rapidly elongating apical part was not significantly different from that in the slow elongating median part, and the osmolarity in the nonelongating basal part was slightly higher than that in the apical and median part. The relationship between osmolarity, osmotic stress, and HOG pathway activation in the stipe of fruiting body needs to be further analyzed in future studies. A higher H_2_O_2_ concentration in the rapidly elongating apical part meant higher amounts of ROS and higher oxidative stress in this region than in the median and basal part of the stipe [65,66,67]. As the relationship between the HOG pathway and light in fungi has been reported [28,29], the fruiting body development of SakA mutants under different light conditions was also investigated. However, no phenotypic differences were found between the SakAi, SakAm, and mock transformants. The results indicated that the HOG pathway of *C. cinerea* may respond to oxidative stress in the elongating part of the stipe, and then, the MAPK of the HOG pathway of *C. cinerea*, CcSakA, was activated to regulate the expression of CcChiE1.

## Data Availability

Not applicable.

## Supplementary Materials

The following supporting information can be downloaded at: https://www.mdpi.com/article/10.3390/jof8050534/s1, Table S1: Oligonucleotides used in this study; Figure S1: The chitinase activity of CcSakA gene silencing (SakAi) transformants, CcSakA phosphomimicking mutant (SakAm) transformants, and mock transformants.
