# Supplementary material for: MAPK CcSakA of the HOG Pathway Is Involved in Stipe Elongation during Fruiting Body Development in Coprinopsis cinerea
Source: J Fungi (Basel). 2022 May 20;8(5):534. doi: 10.3390/jof8050534 (PMC9147448; doi:10.3390/jof8050534)
Supplement: Supplementary file 1 [file jof-08-00534-s001.zip › jof-1635461-supplementary.pdf]

Table S1. Oligonucleotides used in this study

| Name | Sequence (5'-3') | Tm (°C) |
|---|---|---|
| Primers used for construction of plasmids | | |
| SakAi-AS-F | TACACACAACAAGCTCATCGCCATGGGATACGAGCCAATCCGAAATCG | 63.5 |
| SakAi-AS-R | GTTCGTTGGCAATACTCCACCCATGGCGAGTACAATGTCCTTCGTCAAAC | 60.8 |
| SakAi-S-F | GGTGCACGGGAATATTTCGCGGTACCCGAGTACAATGTCCTTCGTCAAAC | 60.8 |
| SakAi-S-R | GATCCCGGTCGGCATCTACTGGTACCGATACGAGCCAATCCGAAATCG | 63.5 |
| SakAm-1-F | TACACACAACAAGCTCATCGCCATGTCCTTCGTCAAACTCAGCAT | 60.5 |
| SakAm-1-F | AGACATCGCCCTCCATTTGCGGATCCTGGATACG | 82.9 |
| SakAm-2-F | AATGGAGGGCGATGTCTCGACACGATACTACCGGG | 82.2 |
| SakAm-2-R | CGGTCGGCATCTACTGGTACCTCACTTATCGTCGTCATCCTTGTAATCAGCATGGCCATTCGAGTAGC | 85.6 |
| Primers used for Vector verification of different transformants | | |
| pCC-SakAi1-F | CGAAGAAGAATTAAGAGGTCCGCAA | 65.4 |
| pCC-SakAi1-R | TACTTTGTCACAGAGCTCCTCGGAA | 64.5 |
| pCC-SakAi2-F | ACTTTGTCACAGAGCTCCTCGGAAC | 65.2 |
| pCC-SakAi2-R | CGTACTAGGGTTGCGAGGTCCA | 64 |
| pCC-SakAm1-F | TGTTCTCGGTGTTTAGGGGTTAGCA | 66.2 |
| pCC-SakAm1-R | CGAGGACCGGTGTGCTGAAAG | 65.1 |
| pCC-SakAm2-F | AAGCGGAAGAGAAATTTGATTGGAG | 64.6 |
| pCC-SakAm2-R | TCTGGAAGAGGTAAACCCGAAACG | 65.8 |
| pCC-ck1-F | GGGCTGGCTTAACTATGCGGCATC | 69.9 |
| pCC-ck1-R | AGATGGTGGATGTGACCGGAATTGG | 69.9 |
| pCC-ck2-F | CGAGACTGAGGAATCCGCTCTTGGC | 71.3 |
| pCC-ck2-R | TCCGGCTCGTATGTTGTGTGGAATTG | 70.8 |
| pab1-ck1-F | CAGGAAACAGCTATGACCATGATTACGC | 68.4 |
| pab1-ck1-R | GCGTGAATGAGTCGTACGAATCGAC | 67 |
| pab1-ck2-F | GGTGAGGAAGTTGAGGTCGGTATGG | 67.1 |
| pab1-ck2-R | GTAAAACGACGGCCAGTGAATTGTAATAC | 66.9 |
| Primers used in qRT-PCR | | |
| Q-β-tubulin-F | GGAGAGACCTTTTGGGAGATGC | 61.8 |
| Q-β-tubulin-R | CATGGTCGACTTGGTCGAAATATAC | 62.1 |
| Q-SakA-F | GCTCCTCGGAACTGACCTTCAC | 62.6 |

| | | |
|---|---|---|
| Q-SakA-R | CGACACCCGCTGAATGAACG | 64.4 |
| Q-ChiE1-F | TACGGCTTGGACGGTCTGGA | 63.5 |
| Q-ChiE1-R | TCCTTCGCCGTTCTCCCTCT | 63.4 |
| Q-ChiIII-F | GCCAACCACCAGAAGCGACT | 62.3 |
| Q-ChiIII-R | TGGCGAGGTTGATGGATGGT | 63.1 |
| Q-Chis1-F | AGTTCGCTGGTGGCTTCTAC | 57 |
| Q-Chis1-R | GCCTCCTCCACTCTCTCTCA | 56.3 |
| Q-Chis2-F | CAGGCGGCTTTAGGGACAAT | 61.7 |
| Q-Chis2-R | CGGGTACATCAAGGACGAGG | 60.1 |

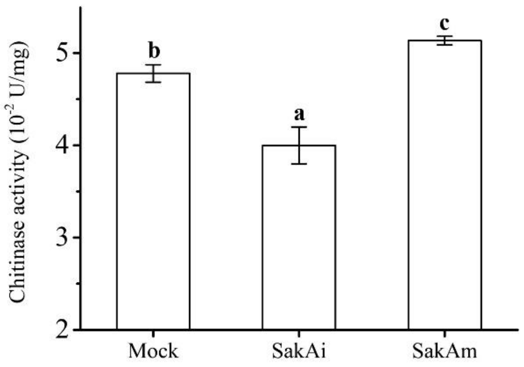


Figure S1. The chitinase activity of CcSakA gene silencing (SakAi) transformants, CcSakA phosphomimicking mutant (SakAm) transformants and mock transformants (n=9). The different letters (a, b and c) indicate significant differences ($P < 0.05$) by Duncan’s test.
